# Exploring access to genomic risk information and the contours of the HIPAA public health exception

**DOI:** 10.1093/jlb/lsac034

**Published:** 2022-12-10

**Authors:** Jennifer K Wagner, Juhi K Tanniru, Courtney A Chane, Michelle N Meyer

**Affiliations:** School of Engineering Design and Innovation; Department of Biomedical Engineering; Penn State Law; Rock Ethics Institute; Institute for Computational and Data Science; Huck Institutes of the Life Sciences, Pennsylvania State University, University Park, PA, USA; Pennsylvania State University, University Park, PA, USA; Pennsylvania State University, University Park, PA, USA; Center for Translational Bioethics and Health Care Policy; Steele Institute for Health Innovation, Geisinger, Danville, PA, USA

**Keywords:** Precision public health, Public Health Genomics, HIPAA, Public Health, Privacy, Genetic Testing

## Abstract

Considerable resources have been invested in research to identify pathogenic and likely pathogenic variants that cause morbidity and mortality and also in returning these results to patients. The public health impact and cost-effectiveness of these efforts are maximized when probands’ relatives are informed of their risk and offered testing. However, such ‘Traceback’ cascade testing programs face multiple obstacles, including perceived or actual legal and regulatory hurdles. Here, using genetic cancer syndromes as a test case, we explore the contours of the Public Health Exception to the HIPAA Privacy Rule to assess whether it is a viable pathway for implementing a Traceback program. After examining the Privacy Rule as well as state laws and regulations for reportable conditions and genetic privacy, we conclude that this is not currently a viable approach for Traceback programs. We conclude by reflecting on ethical considerations of leveraging HIPAA’s public health exception to disclose PHI directly to at-risk relatives and offering insights for how legal hurdles to such a Traceback program could be overcome, if desired.

## I. INTRODUCTION

Improving access to genomic risk information has been a growing priority over the past 20 years, with efforts advancing in both medicine and public health domains and the emergence of ‘precision medicine’^1^ and ‘precision public health.’^2^ The population health impact and cost-effectiveness of these programs are premised on ‘Traceback’ cascade testing, in which at-risk first-degree relatives of probands (patients with a pathogenic or likely pathogenic variant) are notified of their risk and invited for genetic testing themselves.^3^ However, genetic counselors have expressed frustration with low uptake of Traceback testing. One barrier to uptake is that Traceback programs traditionally run through the proband; that is, the proband is encouraged to communicate their relatives’ risk and testing recommendation to them. But probands do not always appreciate the importance of communicating familial risks to relatives promptly, are sometimes apprehensive about having such conversations with their relatives on their own, and are sometimes simply unwilling (for a variety of reasons) to share their genetic risk information with others.

Yet genetic counselors have reported uncertainty about whether and how they may communicate genetic risk information *directly* to at-risk relatives. A particularly acute example of this general problem is Traceback screening for ovarian cancer—a condition for which germline mutations in *BRCA1* and *BRCA2* are known to increase one’s risk and for which multiple current clinical guidelines recommend genetic testing.^4^ Unfortunately, ovarian cancer is often detected too late to effectively treat. This means both that genetic testing of at-risk relatives is critical to support early intervention and that probands often die before Traceback testing can be broached, adding to the list of reasons above why an approach to cascade testing that runs through the proband is problematic.

Scholars have recently clarified the ability of healthcare providers to facilitate expanded access to genomic risk information by assisting the communication of familial risks between a proband and the proband’s relatives; directly contacting at-risk relatives with the proband’s authorization; or contacting the healthcare provider of the proband’s relatives.^5^ The Health Insurance Portability and Accountability Act^6^ (HIPAA) Privacy Rule,^7^ which sets a federal floor of privacy protections for patients’ health information^8^ and usually preempts conflicting state law,^9^ generally allows for protected health information (PHI) to be accessed and used ‘to carry out treatment, payment, or health care operations’ without any specific prior authorization from patients to do so.^10^ Moreover, according to well-established guidance from the Department of Health and Human Services (DHHS),^11^ ‘treatment’^12^ purposes can involve not only the treatment of that one specific patient but also other patients (such as at-risk family members to facilitate cascade genetic screening). Some scholars therefore have interpreted the HIPAA Privacy Rule as permitting *limited* forms of a Traceback program. Specifically, they conclude that HIPAA permits providers to disclose a proband’s PHI either directly to at-risk relatives of the proband (with the patient proband’s authorization) or indirectly to another healthcare provider who is treating the at-risk relative (without the proband’s authorization).^13^

An open question, however, is whether HIPAA permits providers to *directly* contact at-risk relatives without the proband’s authorization. Prior scholarship has dismissed this possibility without closely scrutinizing the relevant text of the HIPAA Privacy Rule that might be most useful for advancing precision public health by allowing direct contact of at-risk relatives even in instances in which no proband authorization is available: the Public Health Exception (PHE) to the HIPAA Privacy Rule. Here, we explore the contours of the PHE as it might relate to Traceback programs, using genetic cancer syndromes as a test case.

## II. THE HIPAA PRIVACY RULE’S PUBLIC HEALTH EXCEPTION (PHE)

The HIPAA Privacy Rule contains a PHE allowing healthcare providers to ‘use or disclose protected health information for public health activities and purposes’ without having to seek the specific patient’s permission.^14^ The exception enumerates to whom healthcare providers may share such information, specifically allowing the disclosures to

[a] public health authority that is authorized by law to collect or receive such information for the purpose of preventing or controlling disease, injury, or disability, including but not limited to, the reporting of disease, injury, vital events such as birth or death, and the conduct of public health surveillance, public health investigations, and public health interventions…^15^

or directly to

[a] person who may have been exposed to a communicable disease or may otherwise be at risk of contracting or spreading a disease or condition, if the covered entity or public health authority is authorized by law to notify such person as necessary in the conduct of a public health intervention or investigation…^16^

Given the aforementioned text, the HIPAA PHE theoretically would enable healthcare providers (such as genetic counselors) to overcome hurdles in the cascade testing process and either (i) disclose to a ‘public health authority’ that is in turn ‘authorized by law’ to disclose to relatives or (ii) disclose directly to at-risk relatives as an employee of a covered entity ‘authorized by law’ to do so as part of ‘a public health intervention or investigation’—in either case, even when the patient-proband is recently deceased^17^ and unable to give consent^18^; is not reachable for whatever reason; or holds steadfast genetic privacy preferences and objects to the disclosure of the information to their at-risk relatives.

Although HIPAA defines ‘public health authority’ as

an agency or authority of the United States, a State, a territory, a political subdivision of a State or territory, or an Indian tribe, or a person or entity acting under a grant of authority from or contract with such public agency, including the employees or agents of such public agency or its contractors or persons or entities to whom it has granted authority, that is responsible for public health matters as part of its official mandate,^19^

there has been some regulatory confusion—magnified by the COVID-19 pandemic—as to what constitutes public health *activities* (as opposed to public health *research*) and which persons or entities may lawfully carry out such public health activities.^20^ The conceptualization of a ‘public health authority’ is irrefutably broad;^21^ however, public health surveillance activities are expressly defined as non-research and therefore excluded from research regulatory oversight,^22^ although it is perhaps intended to be narrowly construed.^23^

Although public health *research* is not the focus of this exploration of the feasibility of a Traceback program for genetic cancer syndromes and how ‘public health authority’ is interpreted there does not necessarily compel us to adopt the same interpretation under the HIPAA Privacy Rule, a brief look at recent discussions is illuminating. In late July 2020, the Secretary’s Advisory Council on Human Research Protections (SACHRP) weighed in on the interpretation of ‘public health authority’ and ‘public health surveillance activities’ within the context of the 2018 Common Rule Requirements^24^ for research involving human participants and offered the Office of Human Research Protections some recommendations.^25^ SACHRP generally recommended a narrow interpretation and application of the public health exclusion from the research regulations, explaining that public trust could be eroded and discouraging broad application ‘outside of a public health emergency’ context.^26^ SACHRP expressed its expert opinion that a person or entity (including private companies, academic institutions, or others) may be considered a ‘public health authority’ for research regulatory purposes^27^ but emphasized the importance of that person or entity acting pursuant to a documented delegation of legal authority (based in governmental statutes or regulations and memorialized by a document such as a memorandum of understanding (MOU), contract, purchase order, or letter that carefully defines the activity) by the public health authority to act on its behalf.^28^ SACHRP further indicated that public health surveillance activities may be ‘passive’ or ‘active’ (such as the notifiable disease reporting system or the Active Bacterial Core System, respectively).^29^ To determine whether a project is a public health surveillance activity that is excluded from the 2018 Common Rule Requirements, SACHRP indicated the following questions must be answered affirmatively: (a) ‘Is the project conducted, supported, requested, ordered, required, or authorized by a public health authority?’; (b) ‘Does the project involve public health surveillance activities, including the collection and testing of information or biospecimens?’; (c) ‘Does the project involve only public health surveillance activities?;’ and (d) ‘Are the public health surveillance activities limited to those necessary to allow a public health authority to identify, monitor, assess, or investigate public health signals, onsets of disease outbreaks, or conditions of public health importance (including trends, signals, risk factors, patterns in diseases, or increases in injuries from using consumer products)?’^30^

This emphasized scrutiny of the nexus or partnership between biomedical professionals and public health authorities discussed in the context of research regulations is relevant to our exploration of whether the HIPAA PHE is viable for a Traceback program. Whether the conditions of the HIPAA PHE at 45 C.F.R. 164.512(b)(i) and 45 C.F.R. 164.512(b)(iv) would be satisfied and thus enable direct disclosure of genetic risk information to a proband’s at-risk relatives for carrying out a Traceback program for genetic cancer syndromes requires a finding that the disclosure is a ‘necessary’ part of a ‘public health intervention or investigation’ as well as ‘authorized by law,’ and evidence for such a finding theoretically would include similar documentation (e.g., memoranda of understanding) between the healthcare providers and public health authority.

What does the HIPAA PHE’s reference to ‘authorized by law’ mean? Where would genetic counselors or other genomic medicine professionals look if they are curious about whether they may lawfully communicate genetic risk information directly to a proband’s relative who might be at risk of developing a genetic disease or condition and who might be at risk of passing along (i.e., ‘spreading’) a genetic disease or condition to others (namely, the next generation)? Does the HIPAA PHE’s language indicating that direct disclosures be to those individuals who ‘may otherwise be at risk of contracting or spreading a disease or condition’ encompass heritable risks of genetic conditions such as breast and ovarian cancers?

In addition to examining the text of the PHE to the HIPAA Privacy Rule, some might also look to legislative or regulatory history for reassurance that applying the PHE to genomic conditions would not clearly run counter to policymakers’ intent. When HIPAA was enacted in 1996, Congress included a provision in the statute that required DHHS to promulgate a privacy rule if Congress itself was unable to pass health information privacy legislation before August 1999. Congress did not meet this self-imposed deadline, and DHHS proceeded accordingly. A proposed rule was announced in November 1999,^31^ and DHHS considered > 50,000 public comments before issuing its final rule 1 year later.^32^ In its final rule, DHHS referred to ‘genetic’ or ‘hereditary information’ dozens of times, devoted a section to ‘Advances in Genetic Sciences,’^33^ and specifically considered privacy matters related to possible disclosures of ‘genetic and hereditary information [of deceased individuals] on living individuals’^34^ and the relevance of ‘information about illnesses with a genetic component’ for the treatment of others.^35^

Yet although DHHS therefore clearly anticipated the applicability of HIPAA to genetic information, it rejected characterizations of the PHE as ‘an inappropriately broad loophole’^36^ and suggestions that the Privacy Rule should impose limits on public health functions, explaining that the PHE was kept sufficiently narrow by Congress, given that such activities must be ‘established by law.’^37^ Although DHHS clarified the Privacy Rule by adding a definition of what is ‘required by law,’^38^ it did not add a new definition for either ‘established by law’ or ‘authorized by law.’ Moreover, DHHS explicitly declined ‘to distinguish between disclosure of information about communicable diseases and disclosure of genetic information’ in ‘allow[ing] disclosure of protected health information to health care providers for purposes of treatment, including treatment of persons other than the individual.’^39^

Nor, when DHHS has had opportunity to modify the HIPAA Privacy Rule, has it taken action to narrow the PHE or single out genetic information for exceptional treatment. For example, in 2002, when DHHS considered modifications to the PHE specifically,^40^ the agency did not revisit the provision allowing for disclosures directly to at-risk individuals.^41^ More than a decade later with promulgation of the Omnibus Rule^42^ to modify the Privacy Rule to implement the HITECH Act^43^ and GINA,^44^ there is no documentation to support the notion that policymakers distinguished between horizontal and vertical transmissions of conditions or were interested in modifying the rule to preclude genetic risk information from being disclosed under the PHE if such disclosures were otherwise authorized by law. In fact, although ‘[o]ne commenter was opposed to the public health exception altogether, stating that it is a privacy loophole that eliminates consumer control over their protected health information,’^45^ DHHS actually added a new provision to the PHE that allowed disclosures of student immunization to schools.^46^

As our examination of the text and legislative/regulatory history reveals, the HIPAA PHE works in a permissive way, allowing but not obligating healthcare professionals to make disclosures. However, unlike other exceptions to the HIPAA Privacy Rule that hinge on whether disclosures are ‘required by law,’ the HIPAA PHE hinges on whether the notification has been ‘authorized’ by law as part of a ‘public health intervention or investigation’ (a conditional threshold that could be met if a public health law permits but does not mandate such disclosures to occur). Thus, the HIPAA Privacy Rule might be a viable permissive pathway for implementation of Traceback programs if distinct laws authorize notification for the genetic conditions as part of public health activities.

## III. APPLICABILITY OF THE HIPAA PHE TO GENOMICS

Public health activities typically are a function of specific state and local—not federal—laws for reportable conditions, and these regulatory frameworks impose information disclosure requirements on healthcare providers to state or local health departments. As a result, the specific conditions for which a healthcare provider might be authorized to disclose as reportable health condition and corresponding information could vary from one jurisdiction to another. At a national level, public health surveillance is performed through the Centers for Disease Control through its reliance on the National Notifiable Diseases Surveillance System (or NNDSS), with the Council of State and Territorial Epidemiologists (CSTE) determining each year which conditions are nationally notifiable.^47^

Efforts to deploy genomics as a public health initiative have been underway for decades.^48^ Pilot programs (such as one deployed in Michigan 2003–2008)^49^ have explored integration of genomic information for prevention of chronic diseases (such as diabetes, asthma, and heart disease) and also have explored whether public health genomics (for conditions such as sudden cardiac death and cancer) could be successfully implemented if public health authorities were to collect more detailed family history data (such as through the Behavioral Risk Factor Surveillance System, BRAFSS, surveys)^50^ or directly employ genetic counselors to carry out genetic screening.^51^ Although these pilot programs have highlighted the potential benefits of healthcare providers and public health authorities collaborating more closely in these efforts (including specifically for hereditary cancer surveillance^52^), they also identified considerable obstacles that have likely not been abated (e.g., lack of financial resources not only to support competitive salaries to recruit and retain adequate genetic counseling expertise to implement a public health genomic program but also to cover associated costs to individuals who are uninsured, under-insured, or receiving Medicaid).^53^ To our knowledge, however, there has not been a recent or systematic examination as to whether specific genetic conditions are already present on reportable or notifiable conditions lists to be leveraged in this manner, and both scholars and practitioners report that they are unaware of how such a process might work.

To explore the feasibility of a genetic Traceback program relying upon HIPAA PHE authorizations, we conducted a 50-state survey^54^ to cross-check whether genetic conditions were already present on the state reportable conditions lists and the NNDSS list. To do so, we used two distinct general public-facing lists to identify which conditions would be considered ‘genetic’: a list posted prominently on the National Human Genome Research Institute (NHGRI) website^55^ and the conditions included in the Genetic Home Reference (GHR), a resource that is now part of MedlinePlus.^56^ The former can be considered to be a conservative list and the latter a more liberal list of genetic conditions. We first checked the NNDSS list to see if those conditions appearing on the NHGRI or GHR lists were nationally notifiable.^57^ Subsequently, we checked whether the conditions appearing on each state’s reportable conditions list were considered to be a genetic condition as per the NHGRI or GHR lists.^58^

The NNDSS list was found to include two conditions appearing on the NHGRI list (i.e., cancer and Kawasaki syndrome) and six conditions appearing on the GHR list as genetic (i.e., cancer, encephalitis, Hansen’s disease or Leprosy, hemolytic uremic syndrome or HUS, Kawasaki syndrome, and Lyme’s disease). As shown in Table 1a, eight conditions that are listed on the NHGRI website as being a genetic condition were observed on one or more state reportable conditions lists: Cancer (including breast cancer, colon cancer, prostate cancer, and skin cancer), Biotinidase deficiency, Congenital hypothyroidism, Galactosemia, Maple syrup urine disease, Phenylketonuria, Sickle Cell Disease (newborns), and Hemophilia. As shown in Table 1b, 15 conditions that are on the GHR list as having a genetic basis were observed on one or more state reportable conditions lists: Biotinidase Deficiency, Cancer, Congenital Anomalies, Congenital Hypothyroidism, Galactosemia, Guillain-Barre Syndrome, Hansen’s disease (Leprosy), Hemophilia, Kawasaki Syndrome, Lyme Disease, Maple Syrup Urine Disease, Phenylketonuria, Pulmonary Fibrosis, Sickle Cell Disease (newborns), and Transmissible Spongiform Encephalopathies (Prion Diseases).

**Table 1:** Reportable Conditions Lists Sometimes Include Cancer and Other Genetic Conditions

**a.**		**b.**	
**‘Genetic Conditions’ as per NHGRI**	**States where reportable**	**‘Genetic Conditions’ as per GHR**	**States where reportable**
Breast cancer	CA^*^, ID, FL^**^, LA, PA, TX, WV, WY	Cancer	CA, ID, FL, LA, PA, TX, WV, WY
Colon cancer	CA^*^, ID, FL^**^, LA, PA, TX, WV, WY		
Prostate cancer	CA^*^, ID, FL^**^, LA, PA, TX, WV, WY		
Skin cancer	CA^*^, ID, FL^**^, LA, PA, TX, WV, WY		
Hemophilia	LA	Hemophilia	LA
Phenylketonuria	ID, LA, PA	Phenylketonuria	ID, LA, PA
Sickle cell disease	LA	Sickle cell disease	LA
		Biotinidase Deficiency	ID
		Congenital Anomalies	FL
		Congenital Hypothyroidism	ID, LA
		Galactosemia	ID, LA
		Guillain-Barre syndrome	MI, PA, VT
		Hansen’s disease or Leprosy	AL, AK, AZ, AR, CA, CO, CT, DE, FL, GA, HI, ID, IN, IA, KS, KY, LA, MD, MA, MI, MS, MO, MT, NE, NV, NH, NJ, NM, NC, OH, RI, SC, SD, TN, TX, UT, VA, WI, WY, AS, GU, VI
		Kawasaki syndrome	DE, MD, MI, MN, NE, WI, GU
		Lyme disease	AL, AK, AZ, AR, CA, CO, CT, DE, FL, GA, ID, IL, IN, IA, KS, KY, LA, ME, MD, MA, MI, MN, MS, MO, MT, NE, NV, NH, NJ, NM, NY, NC, ND, OH, OK, OR, PA, RI, SC, SD, TN, TX, UT, VT, VA, WA, WV, WI, WY, DC, GU, VI
		Maple syrup urine disease	ID
		Pulmonary fibrosis	NY
		Transmissible Spongiform Encephalopathies (Prion Diseases)	AK, ID, MT, RI, UT, WA

^*^Excluding (1) basal and squamous skin cancer unless occurring on genitalia, and (2) carcinoma in-situ and CIN III of the Cervix.

^**^Excluding non-melanoma skin cancer.

Revisiting the text of the HIPAA PHE that permits disclosure to those individuals who might ‘otherwise be at risk of contracting or spreading a disease or condition,’ the appearance of genetic conditions on state reportable conditions lists arguably supports the interpretation that this exception should generally permit direct contact of healthcare providers for purposes of a cascade testing screening program, at least for those specific conditions and, by analogy, lend support for expanding the interpretation to encompass other genetic conditions important for public health. However, a counterargument is that the conditions listed (such as Leprosy or Lyme’s disease) were included without regard to genetic risk factors or heritability but, rather, due to infectiousness or known pathways for transmission to others (including shared environmental exposures). In addition, there are states in which cases of cancer are reportable by healthcare providers to a cancer registry but disclosures of information to other individuals (including for purposes of disease prevention of at-risk relatives) are not permissible under the current regulatory framework for the registry.

Even if one were to conclude that a healthcare provider is ‘authorized by law’ to disclose for a particular reportable or notifiable condition, this does not end the inquiry for whether a Traceback program is feasible. Because the HIPAA Privacy Rule sets a protective floor and not a ceiling for health information privacy, such disclosures made directly to at-risk relatives might nevertheless be precluded by more specific, privacy-preserving laws, such as state health information privacy laws that are more protective than HIPAA, as well as genetic information privacy statutes.^59^ To examine this issue, a 50-state survey was performed to update the information regarding genetic information privacy laws reported by Prince (2013).^60^

**Figure 1:**
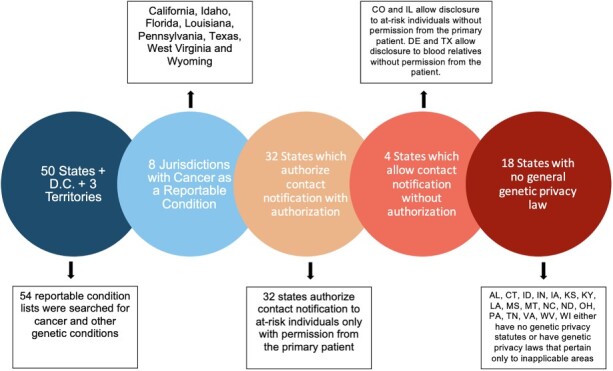
Summary of State Survey Findings.

The genetic privacy laws referenced for each state in Prince (2013) were analyzed for currentness and relevance to Traceback programs. Since 2013, many states have revised and/or added general genetic privacy laws. The use of ‘generic’ privacy laws means the genetic privacy law is not solely pertaining to paternity, insurance, or labor contexts. Table 2 identifies the states that have implemented a genetic privacy law potentially applicable to Traceback programs, healthcare providers, and the extent to which the law allows disclosure to at-risk individuals. As of May 2022, 32 states require authorization from the index patient or proband before any type of disclosure. Only two states, Colorado and Illinois, expressly permit disclosure of disease information to individuals, including at-risk relatives, without permission from the patient. Delaware and Texas allow disclosure to blood relatives without authorization. In the remaining 18 states, there are either no genetic privacy statutes or there are genetic privacy laws that pertain only to inapplicable areas (such as insurance and parentage), and are silent on the topic of disclosure. See Figure 1 summary.

**Table 2:** State-specific genetic information statutes

**State**	**Has a generic genetic privacy statute**	**Allows disclosure of reported disease information to at-risk relatives/individuals only with authorization from the primary person**	**Allows disclosure of reported disease information to at-risk relatives/individuals without authorization from the primary person**	**Allows disclosure of reported disease information to blood relatives without authorization from the primary person**	**Disclosure is up to the discretion of the primary physician**	**Citation**
Alabama						
Alaska	**X**	**X**				Alaska Stat. Ann. § 18.13.010 (West)
Arizona	**X**	**X**			**X**	Ariz. Rev. Stat. Ann. § 12–2802
Arkansas	**X**	**X**				Ark. Code Ann. § 16-43-1101 (West)
California	**X**	**X**				Cal. Civ. Code § 56.181 (West)
Colorado	**X**	**X**	**U**			Colo. Rev. Stat. Ann. § 25–1-122 (West)
Connecticut						
Delaware	**X**	**X**		**U**		Del. Code Ann. tit. 16, § 1205 (West)
Florida	**X**	**X**				Fla. Stat. Ann. § 760.40 (West)
Georgia	**X**	**X**				Ga. Code Ann. § 33–54-3 (West)
Hawaii	**X**	**X**				Haw. Rev. Stat. Ann. § 432:1–607 (West)
Idaho						
Illinois	**X**	**X**	**U**		**U**	410 Ill. Comp. Stat. Ann. 513/15
Indiana						
Iowa						
Kansas						
Kentucky						
Louisiana						
Maine	**X**	**X**			**X**	Me. Rev. Stat. tit. 22, § 1711-C
Maryland	**X**	**X**				Md. Code Ann., Ins. § 27–909 (West)
Massachusetts	**X**	**X**				Mass. Gen. Laws Ann. ch. 111, § 70G (West)
Michigan	**X**	**X**			**U**	Mich. Comp. Laws Ann. § 333.17020 (West)
Minnesota	**X**	**X**				MINN. STAT. ANN. § 13.386(3) (West 2012)
Mississippi						
Missouri		**X**				Mo. Ann. Stat. § 375.1309 (West)
Montana						
Nebraska	**X**	**X**			**U**	Neb. Rev. Stat. Ann. § 71–551 (West)
Nevada	**X**	**X**				Nev. Rev. Stat. Ann. § 629.161 (West)
New Hampshire	**X**	**X**				N.H. REV. STAT. ANN. § 141-H:2 (2012)
New Jersey	**X**	**X**				NJ Rev Stat § 10:5–44 (2019)
New Mexico	**X**	**X**				N.M. Stat. Ann. § 24–21-3 (West)
New York	**X**	**X**				N.Y. Civ. Rights Law § 79-l (McKinney)
North Carolina						
North Dakota						
Ohio						
Oklahoma		**X**				Okla. Stat. Ann. tit. 36, § 3614.3 (West)
Oregon	**X**	**X**			**U**	OR. REV. STAT. ANN. § 192.531(1) (West 2009)
Pennsylvania						
Rhode Island	**X**	**X**				27 R.I. Gen. Laws Ann. § 27–41-53 (West)
South Carolina	**X**	**X**				S.C. Code Ann. § 38–93-40
South Dakota	**X**	**X**				S.D. CODIFIED LAWS § 34–14-22 (2013)
Tennessee						
Texas		**X**		**U**		Tex. Labor Code Ann. § 21.4031 (West) Tex. Labor Code Ann. § 21.403 (West)
Utah	**X**	**X**				Utah Code Ann. § 13–60-202 (West)
Vermont	**X**	**X**				Vt. Stat. Ann. tit. 18, § 9332 (West)
Virginia						
Washington	**X**	**X**			**U**	Wash. Rev. Code Ann. § 70.02.030 (West)
West Virginia						
Wisconsin						
Wyoming	**X**	**X**				Wyo. Stat. Ann. § 35–32-102 (West)

In the chart, ‘X’ indicates that a statute **exists**, and ‘U’ indicates that a statute exists under certain conditions

## IV. DISCUSSION

Our exploration of the PHE to the HIPAA Privacy Rule leads us to conclude that it is not currently a viable approach for Traceback programs. Textual interpretation of the PHE itself is likely to be kept quite narrow (if courts and policymakers apply approaches similar to those used when interpreting other HIPAA Privacy Rule exceptions).^61^ A review of the PHE’s development suggests that policymakers did intend for public health interventions and investigations to be possible regardless of whether the information involved genetic or other types of health information and also intended for such matters to be settled by the external authorizing laws rather than by HIPAA itself. Any given healthcare system interested in pursuing a Traceback program based on the HIPAA PHE would need to perform extensive due diligence to determine and monitor whether state reportable conditions and information privacy laws governing the care of patients in their system (which could involve several states) allow such disclosures.

There are at least three obstacles that would need to be overcome before the PHE is a viable pathway for Traceback programs. Changes to the HIPAA PHE itself are not necessary to make it a viable pathway for Traceback programs, although guidance from DHHS on 45 CFR 164.512(b)(iv) clarifying that disclosure of genetic information if so authorized by state public health law for Traceback programs would not be a HIPAA violation would be welcomed. The first obstacle is clear legislative or regulatory language in state law to authorize public health authorities and healthcare providers to notify at-risk individuals of genetic risk information. This could involve action by the CTSE to add specific genetic conditions to the national notifiable disease list in addition to state legislative action to add specific conditions to each state’s reportable disease lists and to delineate specific implementation guidance for the extent of information that is ‘necessary’ for the precision public health purpose. Reforms of state public health laws to enable Traceback programs for conditions like ovarian cancer would be appropriate on a condition-by-condition basis, but specifications for data minimization to preserve privacy to the extent possible would be critical. The second is reconciling such an authorization with any applicable genetic information privacy laws that might concurrently be in place that impose distinct constraints on uses and disclosures of genetic risk information. Once such ‘authorizations by law’ are in place, a third obstacle to overcome would be for healthcare providing organizations to issue transparent documentation of the precision public health ‘intervention or investigation,’ including the extent to which the healthcare providers are acting independently or as partners with public health authorities.

Of course, *whether* to expand the HIPAA PHE to include genetic conditions for Traceback programs or alternatively to reform state public health laws to authorize use of the HIPAA PHE for Traceback programs for specific genetic conditions is a question not of law, but of ethics and public policy.^62^ When initiating this research, we had three distinct use cases in mind for a possible cancer Traceback program: (i) when a proband has died before the genetic information for the proband’s cancer was available (not uncommon for hard-to-detect, aggressive cancers like ovarian cancer);^63^ (ii) when a proband’s whereabouts are unknown or contact information is not available to provide the proband with an opportunity to authorize or object to a disclosure; and (iii) when a proband has been advised of the importance of notifying at-risk relatives but—for a variety of more or less compelling reasons (e.g., non-paternity versus estrangement)—steadfastly refuses to either do so personally or authorize healthcare providers to do so on the proband’s behalf. We first offer some considerations for and against using a public health framework for Traceback programs that apply to any scenario, and then offer scenario-specific comments.

Regardless of the fact pattern involved, to some skeptics, *ever* treating something like a pathogenic *BRCA1* variant as a public health issue might sound like a category mistake. Distinct frameworks typically apply to clinical ethics and public health ethics—a public health ethics framework often emphasizes solidarity and the public good, whereas clinical ethics often emphasizes individual autonomy^64^—and who has access to a patient’s data might seem like it belongs in the latter category. Yet, although genetic information comes from the proband’s body, it does not pertain to her alone. For instance, when Iceland created a national biobank, the Supreme Court held that a daughter had standing to contest the inclusion of her deceased father’s genetic information, since strong inferences about her could be made on the basis of ‘his’ information.^65^ Relatives can not only be harmed by their relatives’ genetic information, as the Iceland case suggests; they can also benefit—even have their lives saved—as Traceback programs show. More broadly, as the practice of medicine becomes more anticipatory^66^ (rather than merely reactive), health care and public health are coming into closer alignment, with a shared preventative focus and, perhaps, a shared ethics. A similar pattern can be seen in the new concept of the learning health care system,^67^ in which every patient encounter is an opportunity to learn for the benefit of the whole, and low-risk data collection and intervention for learning purposes is frequently done without consent.^68^

On the other hand, to those for whom the idea of a public health approach to genetics is attractive, we note a few cautions. First, although there are compelling reasons for public health, learning health system, and other data surveillance activities to be carried out regardless of an individual’s permissions or objections,^69^ there are differences between traditional public health surveillance and Traceback programs that should give pause. First, and most obviously, although genetic conditions are ‘communicable’ via procreation, they typically do not spread at the scale of many viral diseases.^70^ Disclosing the proband’s genetic information to their relatives without their consent—or over their objections—is also likely to disrupt family relationships. This is not true of many communicable diseases subject to named reporting—though it is notably true of sexually transmitted diseases and other communicable diseases that spread through intimate contact tracing. Genetic information might be regarded as different than other kinds of information,^71^ and it is important to avoid programs that might unreasonably interfere with healthcare access or further undermine public trust in public health measures.^72^ In addition, reframing genetic conditions as akin to ‘infectious diseases’ might exacerbate the guilt that some people already feel upon learning that they have a variant that they might have passed on to their child or other relative.

Finally, it must be acknowledged that genetic privacy^73^ and medical privacy preferences more generally are growing amidst concerns regarding datafication, dataveillance, and data justice^74^ and amidst the chilling effects already observed following the U.S. Supreme Court’s decision in *Dobbs v. Jackson’s Women’s Health Organization*^75^ that abandoned long-standing judicial recognition of a constitutional right to privacy at least as it would pertain to pregnancy termination decisions. With informational privacy law in a constant state of flux and uncertainty, it is unlikely that broadening any exception to the HIPAA Privacy Rule to enable increased data use and disclosures would garner strong political support at this moment in time.

We turn now to the three broad scenarios involving non-consensual disclosure of proband information to at-risk relatives. In the first and second scenarios—the proband is deceased or alive but unreachable through all reasonable means and is not known to object to disclosure—although the privacy interests of the surviving relatives support ongoing duties limiting the use and disclosure of health information even after a proband’s death,^76^ when the information is used in Traceback programs, surviving relatives also stand to directly *benefit* from disclosure of genetic risk information.^77^ If there would have been an ethical duty—based in beneficence and nonmaleficence—to warn the proband in such cases,^78^ arguably the beneficiary of that duty becomes the at-risk relatives when the proband is unavailable. Indeed, some courts have found that a provider’s duty to warn of heritable risks runs to identifiable at-risk relatives.^79^ And when probands are not known to object to disclosure, the health interests of these at-risk relatives likely outweigh the risks of disclosure associated with the proband’s confidentiality interests. However, consistent with broad HIPAA principles, healthcare providers should use and disclose the minimum information necessary to warn at-risk relatives and encourage the uptake of cascade genetic testing.^80^

The third scenario—in which a health system overrides a clear, capacitated ‘no’ from a proband to disclose genetic risk information—is of course the hardest among the fact patterns to ethically justify. Confidentiality and respect for the patient’s autonomy are central to the relationship between a genetic counselor and patient, and unilateral disclosure could undermine trust, cause a contentious and abrupt end to the relationship, and have a chilling effect on other individuals’ willingness to undergo genetic testing of any kind (or, more broadly, to candidly share vital health-related information with providers).

Nevertheless, we agree with the Presidential Commission,^81^ a National Academies committee,^82^ and an American Society for Human Genetics subcommittee^83^ that in some cases, disclosure to at-risk relatives over the objections of the proband is ethically preferable to the alternative. Like these other commenters, we believe whether disclosure is ethically prohibited, permissible, or obligatory depends on multiple factors, including: (a) the magnitude of risk to relatives if they are variant-positive, (b) the degree to which that risk can be mitigated through disclosure, (c) the reasons for the proband’s refusal to disclose, (d) whether disclosure is likely to cause harm (e.g., to the relative, to the proband, to their relationship, or to others), (e) whether the provider could instead point to evidence in the relative’s own medical or social history (as opposed to the proband’s genetic results) to encourage genetic testing, and (f) whether disclosure could be made without revealing the identify of the proband. If the assessment favors disclosure, procedurally, a Traceback program would include steps (i) to document that reasonable efforts to convince a proband to share genomic risk information with the proband’s at-risk relatives have been unsuccessful; (ii) to demonstrate reasonable sensitivity to any known reasons why the proband is resistant to disclosure to at-risk relatives; and (iii) to ensure that the minimum amount of PHI is disclosed to achieve the intended purpose.

Given our analysis of the ethical permissibility (at least in some cases) of a provider disclosing a proband’s genetic results to at-risk relatives under all three scenarios, we therefore believe that the HIPAA Privacy Rule’s PHE should be leveraged ethically and transparently to advance the goals of Traceback programs to reduce morbidity and mortality associated with heritable cancers and many other conditions and diseases. This pathway could help the United States achieve the prioritized goals of the Cancer Moonshot,^84^ which include increasing opportunities for cancer screenings and expanding cancer-prevention approaches to improve health equity.^85^ Ideally, a diverse set of experts would be convened to help identify the specific legal reforms and develop model language (both for satisfying the PHE’s ‘authorized by law’ requirement in state law and for memoranda of understanding, or MOUs, documenting substantive and procedural aspects of public health activities undertaken by healthcare providing organizations) necessary for Traceback programs to be among the strategies pursued to advance prevention, detection, and treatment of cancer. Although institutional and state variation might occur with actual implementation, such guidance would be welcomed and help establish credibility in the endeavor. Such guidance could be developed through roundtable discussions engaging the Centers for Disease Control and Prevention’s Office of Genomics and Precision Public Health; the U.S. Preventative Services Task Force; the National Human Genome Research Institute; the National Cancer Institute; and the newly established Advanced Research Projects Agency for Health (i.e., ARPA-H); members of relevant professional societies (e.g., the American Society of Law, Medicine, and Ethics; the American Public Health Asssociation; the Science & Technology Law and Health Law sections of the American Bar Association; American Law Institute; American Society of Human Genetics; American College of Medical Genetics and Genomics; American Society of Clinical Oncology; American Medical Informatics Association; and American Medical Association); the Council of State and Territorial Epidemiologists; along with patient and community advocates.

Once that occurs, there remain both substantive and procedural design considerations for Traceback programs intended to leverage the HIPAA Privacy Rule’s PHE (reflecting that PHE would be a permissive but not mandatory pathway for disclosure), and the criteria for such Traceback programs should be delineated in institutional policy in advance. For example, although genetic cancer conditions have been our test case for exploring the contours of the PHE, conditions considered for inclusion on a public health reportable/notifiable conditions list should not, in our view, be limited to cancers. Traceback programs should be evidence-based and attuned to equity, so it would be reasonable to encourage inclusion of all CDC Tier 1 conditions.^86^ A recent review has highlighted the benefits of population screening for hereditary breast and ovarian cancer, Lynch syndrome, and familial hypercholesterolemia in particular.^87^ Although the American College of Medical Genetics and Genomics (ACMG) list for reporting secondary findings of clinical exome and genome sequencing is not intended to be a substitute for either diagnostic testing based on clinical criteria or population screening, the medically actionable genes on the current ACMG list^88^ have undergone ample vetting to be considered appropriate for Traceback programs.

Procedural considerations include, for example, setting out steps that healthcare providers must take (as a matter of institutional policy rather than a legal requirement) prior to direct disclosure to the at-risk relatives, particularly if the disclosure is occurring because of active or passive nondisclosure by the proband personally. If health equity is a prioritized feature (as it should be), prior to implementation, Traceback programs also need to take into account the downstream implications for those at-risk relatives (e.g., logistics and out-of-pocket impacts to obtain the genetic testing encouraged; community receptivity to testing; local/state legal protections from discrimination in life, disability, long-term care insurance, and other social settings; etc.)^89^ and devise a strategy for ensuring that, at a minimum, the informational needs can be met.

## V. CONCLUDING REMARKS

Traceback programs are particularly important for addressing ovarian cancer and similar serious genetic conditions. Extensive efforts are underway to identify barriers and facilitators for multi-state Traceback programs, including legal, communication, and other programmatic issues.^90^ At present, those implementing Traceback programs and needing to navigate the complex and ever-changing legal landscape for genetic and health information privacy may, as a starting point, consult this and prior scholarship on acceptable patient-mediated and provider-mediated approaches under HIPAA^91^ and review the LawSeq^SM^ database^92^ and ELSIhub resources.^93^ Even if cascade genetic testing legally must—or for other reasons should—continue to be mediated by the proband, there nevertheless might be ways of improving uptake. For instance, there is some early evidence that technology such as mobile apps^94^ or chatbots^95^ might be useful in increasing uptake of cascade testing. One commonly cited reason why probands refuse to participate in cascade programs is that they are estranged from, or otherwise do not feel comfortable communicating with, the relevant family member(s);^96^ in such cases, these tools’ relative lack of intimacy compares to in-person or telephone conversations might be an advantage. Technology also provides a scalable way to disseminate expert genetic counselor-developed information to at-risk relatives, which can alleviate probands’ concerns that they do not know what to say. ‘Nudges’ and other interventions informed by behavioral science—such as exposing probands to positive stories of cascade testing or to information that most probands find it important to share genetic risk information with their relatives—might also improve uptake.^97^

## Supplementary Material

SupplementalData_lsac034

## Footnotes

1 E.g., A. D. Weston & L Hood, *Systems Biology, Proteomics, and the Future of Health Care: Toward Predictive, Preventative, and Personalized Medicine*, 3 J. Proteome Res. 179–196 (2004); L. A. Hood, Physical Biology: from Atoms to Medicine (Zewail A. H. ed.) 337–366 (Imperial College Press, London, 2008); C. Auffray, D. Charron & L. Hood, *Predictive, Preventive, Personalized and Participatory Medicine: Back to the Future*, Genome Med. 2, 57 (2010); L. Hood & S. Friend, *Predictive, Personalized, Preventive, Participatory (P4) Cancer Medicine*, 8 Nat. Rev. Clin. Oncol. 184–187 (2011); L. Hood & M. Flores, *A Personal View on Systems Medicine and the Emergence of Proactive P4 Medicine: Predictive, Preventive, Personalized and Participatory*, 29 Nat. Biotechnol. 613–24 (2012).

2 E.g., M. J. Khoury, M. S. Bowen, M. Clyne et al., *From Public Health Genomics to Precision Public Health: A 20-Year Journey*, 20 Genet. Med. 574–582 (2018); M J. Khoury, M. F. Iademarco & W. T. Riley, *Precision Public Health for the Era of Precision Medicine*, 50 Am. J. Prev. Med. 398–401 (2016); M. J. Khoury, M. L. Gwinn,R. E. Glasgow, et al., *A Population Approach to Precision Medicine*, 42 Am. J. Prev. Med. 639–645 (2012); M. J. Khoury, *Public Health Genomics: The End of the Beginning*, 13 Genet. Med. 206–9 (2011); M. J. Khoury, W. Burke & E. Thompson, Genetics and Public Health in the 21st Century: Using Genetic Information to Improve Health and Prevent Disease. Oxford University Press: New York, 2000; L. M. Beskow, M. J. Khoury, T. Baker, et al., *The Integration of Genomics into Public Health Research, Policy and Practice in the United States*, 4 Commun. Genet. 2–11 (2001). See also E. Juengst, M. L. McGowan, J. R. Fishman, et al., *The Ethical and Social Implications of Rhetorical Reform in Genomic Medicine*, 46 Hastings Center Rep. 21–22 (2016).

3 U. Ladabaum, G. Wang, J. Terdiman, et al., *Strategies to Identify the Lynch Syndrome among Patients with Colorectal Cancer: A Cost-Effectiveness Analysis*, 155 Ann. Intern. Med. 69–79 (2011).

4 G. Samimi, M. Q. Bernardini, L. C. Brody, et al., *Traceback: A Proposed Framework to Increase Identification and Genetic Counseling of BRCA1 and BRCA2 Mutation Carriers Through Family-Based Outreach*, 35 J. Clin. Oncol. 2329–2337 (2017). doi: 10.1200/JCO.2016.70.3439; D. M. Eccles, J. Balmaña, J. Clune J, et al*., Selecting Patients with Ovarian Cancer for Germline BRCA Mutation Testing: Findings from Guidelines and a Systematic Literature Review*, 33 Adv. Ther. 33 129–150 (2016). doi: 10.1007/s12325-016-0281-1; NCCN Clinical practice guidelines in oncology. Genetic/familial high risk assessment: breast and ovarian version 2.2019. NCCN Guidelines. 2018. Available at https://www2.tri-kobe.org/nccn/guideline/gynecological/english/genetic_familial.pdf; J. M. Lancaster, C. B. Powell, L. M. Chen, et al., *SGO Clinical Practice Committee. Society of Gynecologic Oncology Statement on Risk Assessment for Inherited Gynecologic Cancer Predispositions*, 136 Gynecol. Oncol. 3–7 (2015). doi: 10.1016/j.ygyno.2014.09.009. Epub 2014 Sep 17. Erratum in: Gynecol. Oncol. 2015 Sep;138(3):765; I. Vergote, S. Banerjee, A. M. Gerdes, et al., *Current Perspectives on Recommendations for BRCA Genetic Testing in Ovarian Cancer Patients,* 69 Eur. J. Cancer. 127–134 (2016). doi: 10.1016/j.ejca.2016.10.006; K. A. Metcalfe, I. Fan, J. McLaughlin, et al., *Uptake of Clinical Genetic Testing for Ovarian Cancer in Ontario: A Population-Based Study*, 112 Gynecol Oncol. 68–72 (2009). doi: 10.1016/j.ygyno.2008.10.007; R. Demsky, J. McCuaig, M. Maganti, et al., *Keeping it Simple: Genetics Referrals for all Invasive Serous Ovarian Cancers,* 130 Gynecol. Oncol. 329–333 (2013). doi: 10.1016/j.ygyno.2013.05.003; J. McGee, K. Panabaker, S. Leonard, et al., *Genetics Consultation Rates Following a Diagnosis of High-Grade Serous Ovarian Carcinoma in the Canadian Province of Ontario*, 27 Int. J. Gynecol. Cancer 437–443 (2017). doi: 10.1097/IGC.0000000000000907.

5 Nora B. Henrikson, Jennifer K. Wagner, Heather Hampel, et al., *What Guidance Does HIPAA Offer to Providers Considering Familial Risk Notification and Cascade Genetic Testing?*J. Law Biosci. 2020, lsaa071, https://doi.org/10.1093/jlb/lsaa071.

6 Health Information Portability and Accountability Act of 1996, Pub. L. 104–191, 110 Stat. 1936, as codified at 45 C.F.R. §§160, 162, and 164.

7 45 C.F.R. 164.512(b)(1)(iv).

8 45 C.F.R. Part 160 and Subparts A and E of Part 164. HIPAA/HITECH Omnibus Final Rule is 78 FR 5694 (January 25, 2013).

9
*See infra* note 59.

10 45 C.F.R. 164.506.

11 Office for Civil Rights, *Under the HIPAA Privacy Rule, may a health care provider disclose protected health information about an individual to another provider, when such information is requested for the treatment of a family member of the individual?*, US Department of Health & Human Services (2009), *available at*https://www.hhs.gov/hipaa/for-professionals/faq/512/under-hipaa-may-a-health-care-provider-disclose-information-requested-for-treatment/index.html.

12 45 C.F.R. 164.501 (defining treatment as ‘the provision, coordination, or management of health care and related services by one or more health care providers, including the coordination or management of health care by a health care provider with a third party; consultation between health care providers relating to a patient; or the referral of a patient for health care from one health care provider to another.’).

13 See Scenarios 3 and 6 in Table 1 of Henrikson et al. 2020 (*supra* note 5).

14 45 C.F.R. 164.512(b).

15 45 C.F.R. 164.512(b)(i).

16 45 C.F.R. 164.512(b)(iv).

17 I.e., within 50 years, such that post-death HIPAA Privacy protections have not yet expired. See, e.g., https://www.hhs.gov/hipaa/for-professionals/faq/1500/do-hipaa-protections-apply-to-the-health-information-of-individuals/index.html.

18 Personally or through a proxy (i.e., the executor/executrix or administrator of the decedent’s estate).

19 45 C.F.R. 164.501.

20 See, e.g., Megan Doerr & Jennifer K. Wagner, *Research Ethics in a Pandemic: Considerations for the Use of Research Infrastructure and Resources for Public Health Activities,* 7 J. Law Biosci. lsaa028 (2020), https://doi.org/10.1093/jlb/lsaa028.

21 For example, the National Institutes of Health (NIH) is authorized by law to assist as a ‘public health authority.’ U.S. Dept. of Health and Human Services, Does the HIPAA Privacy Rule’s Public Health Provision Permit Covered Entities to Disclose Protected Health Information to Authorities such as the National Institute of Health (NIH)?, Dec. 12, 2002, https://www.hhs.gov/hipaa/for-professionals/faq/297/does-the-hipaa-public-health-provision-permit-covered-entities-to-disclose-information-to-authorities/index.html (last accessed Aug. 13, 2020).

22 45 C.F.R. §46.102(l)(2); See also U.S. Dept. of Health and Human Services, Office of Human Research Protections, Activities Deemed Not to be Research: Public Health Surveillance 2018 Requirements (reviewed Nov. 12, 2018), https://www.hhs.gov/ohrp/regulations-and-policy/requests-for-comments/draft-guidance-activities-deemed-not-be-research-public-health-surveillance/index.html (last accessed Aug. 13, 2020).

23 45 C.F.R. §46.102(l)(2) articulates in relevant part that the public health surveillance activities are ‘limited to those necessary to allow a public health authority to identify, monitor, assess, or investigate potential public health signals, onsets of disease outbreaks, or conditions of public health importance (including trends, signals, risk factors, patterns in diseases, or increases in injuries from using consumer products)…’.

24 Final Rule: Federal Policy for the Protection of Human Subjects, 82 Fed. Reg. 7149 (Jan. 19, 2017), codified at 45 C.F.R. §46.

25 See, e.g., Meeting of the Secretary’s Advisory Committee on Human Research Protections (SACHRP) July 22–23, 2020, public materials available at Docket HHS-OPHS-2020-0004 in regulations.gov.

26 See ‘SACHRP Recommendations - Draft Public Health Surveillance.7.29.20.Post Meeting_Clean (002)’ (HHS-OPHS-2020-0004-0019) available at https://beta.regulations.gov/document/HHS-OPHS-2020-0004-0019 at page 10.

27 45 C.F.R. 46.102(k) and 46.102(l)(2).

28
*See* ‘SACHRP Recommendations - Draft Public Health Surveillance.7.29.20.Post Meeting_Clean (002)’ (HHS-OPHS-2020-0004-0019) available at https://beta.regulations.gov/document/HHS-OPHS-2020-0004-0019 at pages 3–5.

29
*Id.* at page 8.

30
*Id.* at pages 12–13.

31 DHHS, Proposed Rule: Standards for Privacy of Individually Identifiable Health Information, 64 Fed. Reg. 59,918 (Nov. 3, 1999).

32 DHHS, Final Rule: Standards for Privacy of Individually Identifiable Health Information, 65 Fed. Reg. 82,462 (Dec. 28, 2000).

33 65 Fed. Reg. at 82621.

34 65 Fed. Reg. at 82632.

35 65 Fed. Reg. at 82555, 82631.

36 65 Fed. Reg. at 82623.

37 65 Fed. Reg. at 82624.

38 45 CFR § 164.103 Definitions (defining ‘*Required by law*’ as ‘a mandate contained in law that compels an entity to make a use or disclosure of protected health information and that is enforceable in a court of law…’).

39 65 Fed. Reg. at 82633.

40 DHHS, Final Rule: Standards for Privacy of Individually Identifiable Health Information, 67 Fed. Reg. 53,181 (Aug. 14, 2002).

41 45 CFR 164.512(b)(1)(iv).

42 DHHS, Final Rule: Modifications to the HIPAA Privacy, Security, Enforcement, and Breach Notification Rules Under the Health Information Technology for Economic aand Clinical Health Act and the Genetic Information Nondiscrimination Act; Other Modifications to the HIPAA Rules, 78 Fed. Reg. 5565 (Jan. 25, 2013).

43 Health Information Technology for Economic and Clinical Health Act, Pub. L. 111–5, enacted February 17, 2009.

44 Genetic Information Nondiscrimination Act of 2008, Pub. L. 110–233, 122 Stat. 881.

45 78 Fed. Reg. at 5606.

46 78 Fed. Reg. 5565.

47 For a distinction between ‘reportable’ and ‘notifiable’ conditions, see generally https://www.cdc.gov/nndss/about/index.html (last accessed May 19, 2022). See also https://www.cste.org.

48 E.g., M. J. Khoury, M. S. Bowen, M. Clyne, et al., *From Public Health Genomics to Precision Public Health: A 20-Year Journey*, 20 Genet. Med. 574–582 (2018); M. J. Khoury, M. F. Iademarco & W. T. Riley, *Precision Public Health for the Era of Precision Medicine*. 50 Am. J. Prev. Med. 398–401 (2016); M. J. Khoury, M. L. Gwinn, R. E. Glasgow, et al. *A Population Approach to Precision Medicine*, 42 Am. J. Prev. Med. 639–645 (2012); M J Khoury, *Public Health Genomics: The End of the Beginning,* 13 Genet. Med. 206–209 (2011); M. J. Khoury, W. Burke, E. Thompson, Genetics and Public Health in the 21st Century: Using Genetic Information to Improve Health and Prevent Disease. Oxford University Press: New York, 2000; L. M. Beskow, M. J. Khoury, T. Baker, et al., *The Integration of Genomics into Public Health Research, Policy and Practice in the United States*, 4 Commun. Genet. 2–11 (2001).

49 J. St Pierre, J. Bach, D. Duquette, et al., *Strategies, Actions, and Outcomes of Pilot State Programs in Public Health Genomics, 2003–2008,* 11 Prev. Chronic Dis. E97 (2014). doi: 10.5888/pcd11.130267.

50 J. St Pierre, J. Bach, D. Duquette, et al., *Strategies, Actions, and Outcomes of Pilot State Programs in Public Health Genomics, 2003–2008*, 11 Prev. Chronic Dis. E97 (2014). doi: 10.5888/pcd11.130267.

51 Personal communication between JKW and Debra Duquette on March 23, 2022.

52 See, e.g, Y. T. Yang & B. Chen, *Precision Medicine and Sharing Medical Data in Real Time: Opportunities and Barriers,* 24 Am. J. Manag. Care. 356–358 (2018) (describing a pilot program involving a partnership between the California Department of Public Health and select hospitals to enable real-time surveillance for the California Cancer Registry using a standardized checklist and advocating that states remove barriers for precision public health and facilitate closer connections between public health authorities and healthcare providers).

53 See, e.g., J. St Pierre, J. Bach, D. Duquette, et al., *Strategies, Actions, and Outcomes of Pilot State Programs in Public Health Genomics, 2003–2008*, 11 Prev. Chronic Dis. E97 (2014). doi: 10.5888/pcd11.130267.

54 Our research reviewed all 50 states and the District of Columbia. The US territories were examined to the extent possible. Reportable conditions lists for American Samoa, Guam, and the Virgin Islands were examined, as reported in the supplemental materials; however, because the reportable conditions lists for two of the five territories (i.e., Puerto Rico and Northern Mariana Islands) could not be located or accessed for examination, the results discussed here are focused on the 50 states and District of Columbia.

55 Genetic Disorders, last updated May 18, 2020, https://www.genome.gov/For-Patients-and-Families/Genetic-Disorders (Accessed June 8, 2020).

56 Genetic Conditions, https://ghr.nlm.nih.gov/condition (Accessed June 8, 2022). During this study, the Genetics Home Reference website was discontinued as an independent resource, but ‘most’ information from GHR remains accessible through MedlinePlus and URLs for GHR redirect to MedlinePlus. See *Genetics Home Reference Merged into MedlinePlus*. NLM Tech. Bull. 2020 Sept–Oct; 436: e1 at https://www.nlm.nih.gov/pubs/techbull/so20/so20_ghr_medlineplus_merge.html (which also indicates there are more than 1300 genetics conditions).

57 Cross checks were based on the NNDSS, NHGRI, and GHR lists as they appeared online on June 8, 2020 and on each state’s reportable conditions lists as they appeared during the first half of 2022.

58 Another method for checking reportable conditions across jurisdictions was identified in May 2022. The Council of State and Territorial Epidemiologists (CTSE) has a State Reportable Conditions Assessment (SRCA) searchable database available at http://www.cste.org/page/SRCA; however, searches can only be performed from a pulldown conditions list and only for two time periods: 2007–2010 and 2015–2018. On May 20, 2022, this SRCA was used to explore which jurisdictions had ‘cancer*’ as a reportable condition for the most recent time period. By checking ‘cancer*’ (as the SRCA does not have specific types in the pulldown menu) and ‘healthcare provider’ reporting (as the SRCA offers the option to specify type of reporter: healthcare provider, laboratory, hospital, other, or all), and this identified 31 jurisdictions—AR, CO, CT, DC, FL, GA, IL, IN, KS, LA, MA, MD, MI, MN, MS, MI, MO, ND, NH, NJ, NM, OK, OR, PA, SC, SD, TN, TX, UT, WI, and WY—with explicit cancer reporting requirements for healthcare providers.

59 Although HIPAA generally preempts state laws that are contrary to the Privacy Rule, no preemption exists if it is possible to comply with both laws. Even in a rare case of conflict, a specific exception to HIPAA preemption is for a state law that ‘relates to the privacy of individually identifiable health information and is more stringent.’ 45 C.F.R. 160.203(b).

60 A. Prince, *Comprehensive Protection of Genetic Information: One Size Privacy or Property Models May Not Fit All*. 79 Brooklyn L. Rev. 175 (2013), Available at SSRN: https://ssrn.com/abstract=3064279.

61 Many scholars, practitioners, policy administrators, and members of the public have turned to the HIPAA Privacy Rule urging the need to make disclosures of PHI less likely and to close gaps that currently allow for disclosures. For example, on June 29, 2022, in the aftermath of the *Dobbs v. Jackson’s Women’s Health Organization* decision (No. 19–1392. decided June 24, 2022), the Office of Civil Rights at the U.S. Department of Health and Human Services issued updated guidance on the distinct law enforcement exception to the Privacy Rule, emphasizing how narrow the exception is and deterring healthcare providers from making disclosures pursuant to it without confirming all conditions have been satisfied. See https://www.hhs.gov/hipaa/for-professionals/special-topics/reproductive-health/index.html (last accessed July 6, 2022). Arguments for a broad interpretation of the PHE exception to the Privacy Rule might be disfavored under current sociopolitical conditions, regardless of any public health benefits that might accrue.

62 It is also a matter of organizational risk tolerance. Even in jurisdictions where it appears to be permissible, it would not be required and healthcare organizations would likely differ in their willingness to launch a Traceback program. Leadership of healthcare organizations differ in their attitudes toward preventable cancer-related deaths and their associated costs, as well as their tolerance for legal and public relations risks. Different fact patterns—ranging from disclosure of information of a deceased proband unable to provide authorization to disclosure of information of a living proband over their objections—could influence their risk assessments.

63 See, e.g., D. R. Gordon & B. A. Koenig, ‘*If relatives inherited the gene, they should inherit the data.’ Bringing the Family into the Room Where Bioethics Happens*, 41 New Genet. Soc. 23–46 (2022). doi: 10.1080/14636778.2021.2007065 (discussing the challenges a healthcare system faced following the identification of genetic variants from 75 deceased pancreatic cancer patients who were not asked to provide authorization to disclose information to at-risk relatives and who did not explicitly give consent for familial disclosure prior to their death).

64 C. Klinger, D. S. Silva, C. Schuermann, et al., *Ethical Issues in Public Health Surveillance: A Systematic Qualitative Review*, 17 BMC Public Health 295 (2017); J. F. Childress, R. R. Faden, R. D. Gaare, et al., *Public Health Ethics: Mapping the Terrain*, 30 J. Law Med. Ethics 170–178 (2002); N. Kass, *An Ethics Framework for Public Health*, 91 Am. J. Public Health 1776–1782 (2001); G. R. Swain, K. A. Burns & P. Etkind, *Preparedness: Medical Ethics Versus Public Health Ethics*, 14 J. Pub. Health Mgmt. Pract., 354–357 (2008); L. M. Lee, *A Bridge Back to the Future: Public Health Ethics, Bioethics, and Environmental Ethics*, 17 Am J Bioethics 5–12 (2017).

65
*See* 118 *Harv. L. Rev.*, *infra* note 76.

66 Dr David Feinberg, former CEO of Geisinger, has been quoted as saying, ‘This is what we call “anticipatory medicine”…It means we’re anticipating the medical needs of our patients. We’re finding medically actionable conditions and giving that information back to our patients.’ https://www.geisinger.org/research/research-connections-2/2017/12/12/21/00/more-than-500-mycode-participants-receive-clinical-results-from-exome-sequencing. See also G. Costain, S. Walker, M. Marano, et al. *Genome Sequencing as a Diagnostic Test in Children With Unexplained Medical Complexity*. 3 JAMA Netw. Open. e2018109 (2020). doi: 10.1001/jamanetworkopen.2020.18109 (noting ‘anticipatory care’.

67
*See, e.g.*, Institute of Medicine. Genomics-Enabled Learning Health Care Systems: Gathering and Using Genomic Information to Improve Patient Care and Research: Workshop Summary. Washington (DC): National Academies Press (2015); M. S. Williams, A. H. Buchanan, F. D. Davis, et al., *Patient-Centered Precision Health in a Learning Health Care System: Geisinger’s Genomic Medicine Experience,* 37 Health Affairs. 757–764 (2018). https://doi.org/10.1377/hlthaff.2017.1557.

68
*See, e.g.*, Leora I. Horwitz, Masha Kuznetsoca, Simon A. Jones, *Creating a Learning Health System through Rapid-Cycle, Randomized Testing*, 381 New Eng. J. Med. 1175 (2019).

69
*See, e.g.*, Lisa M. Lee, Charles M. Heilig, & Angela White, *Ethical Justification for Conducting Public Health Surveillance without Patient Consent*, 102 Am. J. PublicHealth 38 (2012). *See also* Nancy E. Kass, *An Ethics Framework for Public Health*, 91 Am. J. PublicHealth 1776 (2001).

70 Interestingly, however, public health’s ‘R number,’ or reproduction number which is often said to be a measure of whether an epidemic is growing or shrinking, has its origins in reproduction and population growth.

71 See, e.g., N. A. Garrison, K. B. Brothers, A. J. Goldenberg, et al., *Genomic Contextualism: Shifting the Rhetoric of Genetic Exceptionalism,* 19 Am. J. Bioeth. 51–63 (2019). doi: 10.1080/15265161.2018.1544304.

72 E.g., Silena Simmons-Duffin, *Poll Finds Public Health Has a Trust Problem*, NPR, 13 May 2021, https://www.npr.org/2021/05/13/996331692/poll-finds-public-health-has-a-trust-problem.

73 Ellen W. Clayton, Colin M. Halverson, Nila A. Sathe, et al., *A Systematic Literature Review of Individuals’ Perspectives on Privacy and Genetic Information in the United States.* 13 PLoS One. e0204417 (2018). https://doi.org/10.1371/journal.pone.0204417.

74 See, e.g., Taylor, Linnet, ‘*What Is Data Justice? The Case for Connecting Digital Rights and Freedoms Globally.*’ 4 Big Data & Society 205395171773633 (2017). https://doi.org/10.1177/2053951717736335; J. Van Dijck, *Datafication, Dataism and Dataveillance: Big Data between Scientific Paradigm and Ideology,* 12 Surveill. Soc. 197–208 (2014). doi: 10.24908/ss.v12i2.4776; M. Buchbinder, E. Juengst, S. Rennie, et al., *Advancing a Data Justice Framework for Public Health Surveillance*, AJOB Empir Bioeth 1–9 (2022). doi: 10.1080/23294515.2022.2063997; A. Kuntsman, E. Miyake & S. Martin, *Re-Thinking Digital Health: Data, Appisation and the (im)possibility of ‘Opting out*’, 9 Digit Health. 2055207619880671 (2019). doi: 10.1177/2055207619880671.

75 No. 19–1392. decided June 24, 2022.

76
*See, e.g.*, Comparative Law. Genetic Privacy. Iceland Supreme Court Holds That Inclusion of an Individual’s Genetic Information in a National Database Infringes on the Privacy Interests of His Child. Guimundsdottir v. Iceland, no. 151/2003 (Nov. 27, 2003) (Ice.), 118 Harv. L. Rev. 810 (2004).

77 See also, e.g., S. Weller, K. Lyle & A. Lucassen*, Re-Imagining ‘the patient’: Linked Lives and Lessons from Genomic Medicine*, 297 Soc. Sci. Med. 114806 (2022). doi: 10.1016/j.socscimed.2022.114806 (discussing nuances of ‘patienthood’ within genomic medicine and the connectedness of individuals intra- and intergenerationally).

78 For debates about provider duties to warn relatives of genetic risks, *see, e.g.*, R. B. Dugan, G. L. Wiesner, E. T. Juengst, et al., *Duty to Warn At-Risk Relatives for Genetic Disease: Genetic Counselors’ Clinical Experience*, 119C Am J. Med. Genet. C Semin. Med. Genet. 27 (2003); B. Godard, T. Hurlimann, M. Letendres, et al., *Guidelines for Disclosing Genetic Information to Family Members: From Development to Use,* 5 Fam. Cancer 103 (2006); S. Dheensa, A. Fenwick, S. Shkedi-Rafid, et al., *Health-Care Professionals’ Responsibility to Patients’ Relatives in Genetic Medicine: A Systematic Review And Synthesis Of Empirical Research*, 18 Genet. Med. 290 (2016); A. Phillips, P. Borry, I. Van Hoyweghen, et al., *Disclosure of Genetic Information to Family Members: A Systematic Review of Normative Documents*, 23 Genet Med. 2038–2046 (2021). doi: 10.1038/s41436-021-01248-0.

79 Safer v. Estate of Pack, 677 A.2d 1188 (N.J. App., appeal denied, 683 A.2d 1163 (NJ 1996)) (holding that the provider has a duty to relatives and must take ‘reasonable steps’ to ensure that they are warned); Pate v. Threlkel, 661 S0.2d 278 (Fla. 1995) (holding that the provider has a duty to warn relatives, but that it is met by warning the proband); Molloy v. Meier, Nos. C9-02-1821, C9-02-1837 (Minn. 2004) (‘a physician’s duty regarding genetic testing and diagnosis extends beyond the patient to biological parents who foreseeably may be harmed by a breach of that duty’).

80
*See also supra* note 63 (criticizing ethical and legal frameworks for excessively privileging individual rights over familial interests and advocating for a ‘joint account model of genomic information’).

81 President’s Commission for the Study of Ethical Problems in Medicine and Biomedical and Biobehavioral Research, Screening and Counseling for Genetic Conditions: The Ethical, Social, and Legal Implications of Genetics Screening, Counseling, an Education Programs (Washington, DC: US Government Printing Office, 1998).

82 Institute of Medicine Committee on Assessing Genetic Risks, Assessing Genetic Risks: Implications for Health and Social Policy (Washington, DC: National Academiy Press, 1994).

83 ASHG Statement. Professional disclosure of familial genetic information. The American Society of Human Genetics Social Issues Subcommmittee on Familial Disclosure. 62 Am. J. Hum. Genet. 474 (1998).

84
https://www.whitehouse.gov/briefing-room/statements-releases/2022/02/02/fact-sheet-president-biden-reignites-cancer-moonshot-to-end-cancer-as-we-know-it/ and https://www.whitehouse.gov/cancermoonshot/ (last accessed Sept. 28, 2022).

85 E.g., https://www.whitehouse.gov/briefing-room/statements-releases/2022/07/13/fact-sheet-president-biden-appoints-cancer-panel-members-and-cancer-cabinet-unveils-priority-actions/ (last accessed Sept. 28, 2022).

86 See also C. G. Allen, R. F. Green, S. Bowen, et al., *Challenges and Opportunities for Communication about the Role of Genomics in Public Health*, 24 Public Health Genomics. 67–74 (2021). doi: 10.1159/000512485; and M. J. Khoury, S. Bowen, W. D. Dotson, et al., *Health Equity in the Implementation of Genomics and Precision Medicine: A Public Health Imperative*, 24 Genet. Med. 1630–1639 (2022). doi: 10.1016/j.gim.2022.04.009 We note that, given Traceback programs’ implications across the lifespan, the factors taken into account when determining whether a condition is ripe for inclusion on a list to be leveraged for Traceback programs might be distinct from those taken into account in determinations as to whether a condition should be included on a newborn screening panel.

87 M. S. Williams, *Population Screening in Health Systems,* 23 Annu. Rev. Genom. Hum. Genet. 549–567 (2022). https://doi.org/10.1146/annurev-genom-111221-115239 (noting at 562 “three conclusions seem evident: (*a*) These three conditions are relatively common hereditary conditions across all populations screened to date; (*b*) current guidelines to identify at-risk individuals relying on personal and family history of disease are insensitive, missing anywhere from 50% to 90% of pathogenic variant carriers; and (*c*) even individuals who meet guidelines for testing are not being uniformly tested, with roughly half of variant carriers not having been offered clinical testing despite meeting testing criteria. These factors lead to lost opportunities for prevent and early intervention that can reduce morbidity and mortality.”).

88 Version 3.1 of the ACMG list includes 78 genes, and the list is now updated annually to add or remove variants from the list. See R. C. Green, J. S. Berg, W. W. Grody, et al., *ACMG Recommendations for Reporting of Incidental Findings in Clinical Exome and Genome Sequencing* [published correction appears in 19 Genet. Med. 606 (2017)], 15 Genet. Med. 565–574 (2013). doi: 10.1038/gim.2013.73; S. S. Kalia, K. Adelman, S. J. Bale, et al., *Recommendations for Reporting of Secondary Findings in Clinical Exome and Genome Sequencing, 2016 Update (ACMG SF V2.0): A Policy Statement of the American College of Medical Genetics and Genomics* [published correction appears in 19 Genet. Med. 484 (2017)], 19 Genet Med. 249–255 (2017). doi: 10.1038/gim.2016.190; D. T. Miller, K. Lee, A. S. Gordon, et al., *Recommendations for Reporting of Secondary Findings in Clinical Exome and Genome Sequencing, 2021 Update: A Policy Statement of the American College of Medical Genetics and Genomics* (ACMG), 23 Genet. Med. 1391–1398 (2021). doi: 10.1038/s41436-021-01171-4; D. T. Miller, K. Lee, N. S. Abul-Husn, et al., *ACMG SF v3.1 List for Reporting of Secondary Findings in Clinical Exome and Genome Sequencing: A Policy Statement of the American College of Medical Genetics and Genomics (ACMG),* 24 Genet Med. 1407–1414 (2022). doi: 10.1016/j.gim.2022.04.006.

89 See, e.g., Samimi et al. *supra* note 4.

90 E.g., A. DiNucci, N. B. Henrikson, M. Caball Jones, et al., F*easability and Assessment of a Cascade Traceback Screening Program (FACTS): Protocol for a Multistate Study to Implement and Assess an Ovarian Cancer Traceback Cascade Testing Program*, 11 J Pers Med. 543 (2021). https://doi.org/10.3390/jpm11060543.

91 Nora B. Henrikson, Jennifer K. Wagner, Heather Hampel, et al., *What Guidance Does HIPAA Offer to Providers Considering Familial Risk Notification and Cascade Genetic Testing?*J. Law Biosci. lsaa071 (2020), https://doi.org/10.1093/jlb/lsaa071.

92
Lawseq.umn.edu (last accessed Aug. 21, 2022).

93
Elsihub.org (last accessed Aug. 21, 2022).

94 C. B. Haas, A. Scrol, C. Jujjavarapu, et al., *Usefuleness of Mobile Apps for Communication of Genetic Test Results to At-Risk Family Members in a U.S. Integrated Health System: A Qualitative Approach from User-Testing,* 10 Health Pol’y Tech. 100,511 (2021).

95 T. Schmidlen, C. L. Jones, G. Campbell-Salome, et al., *Use of a Chatbot to Increase Uptake of Cascade Genetic Testing*, J. Genet. Couns. 2022 doi: 10.1002/jgc4.1592. Advance online publication. https://doi.org/10.1002/jgc4.1592.

96 Dugan et al., *supra* note 78; J. Green, M. Richards, F. Murton, et al., *Family Communication and Genetic Counseling: The Case Of Hereditary Breast And Ovarian Cancer,* 6 J. Genet. Couns. 45 (1997).

97 See, e.g., P. R. Heck &M. N. Meyer, *Population Whole Exome Screening: Primary Care Provider Attitudes About Preparedness, Information Avoidance, and Nudging,* 103 Med. Clin. N. Am. 1077–1092, 1087–1088 (2019).

